# Analyzing web searches for axial spondyloarthritis in Germany: a novel approach to exploring interests and unmet needs

**DOI:** 10.1007/s00296-023-05273-x

**Published:** 2023-01-14

**Authors:** Kristina Berr, Linda Tizek, Maximilian C. Schielein, Martin Welcker, Johannes Knitza, Stefan Kleinert, Alexander Zink

**Affiliations:** 1 Department of Dermatology and Allergy, School of Medicine, Technical University of Munich, Biedersteiner Street 29, 80802 Munich, Germany; 2 Medizinisches Versorgungszentrum für Rheumatologie Dr. M. Welcker GmbH, Planegg, Germany; 3 Department of Internal Medicine 3, Rheumatology and Immunology, Friedrich-Alexander University Erlangen-Nürnberg and Universitätsklinikum Erlangen, Erlangen, Germany; 4 Praxisgemeinschaft Rheumatologie-Nephrologie Erlangen, Rheumatologische Schwerpunktpraxis, Erlangen, Germany; 5 Department of Internal Medicine II, Rheumatology/Clinical Immunology, Universitätsklinikum Würzburg, Würzburg, Germany; 6 Division of Dermatology and Venereology, Department of Medicine Solna, Karolinska Institutet, Stockholm, Sweden

**Keywords:** Axial spondyloarthritis, Web search data, Infodemiology, Burden of disease, Unmet needs

## Abstract

Axial spondyloarthritis (axSpA) is an underdiagnosed condition with a high disease burden. Due to delayed diagnosis and limited access to specialist care, conventional health data might not sufficiently capture the perspective of affected individuals. The aim of this study was to assess public interest, unmet needs, and disease burden of axSpA in Germany through the analysis of thematic, geographic, and temporal patterns in national web search data. Google Ads Keyword Planner was used to identify axSpA-related keywords and their monthly search volume in Germany between January 2017 and December 2020. Identified keywords were qualitatively categorized into six categories. Overall, 265 axSpA-related keywords with a search volume of 3,881,490 queries were identified. Nearly 81% of the total search volume was assigned to the category *terms and definition,* while 19% referred to either *outcomes*, *symptoms*, *diagnosis*, *management*, or *causes*. In the category *outcomes*, *prognostic outcomes* like “life expectancy” generated more searches than *physical manifestations* like “pain”. Less populated cities showed significantly more searches per 100,000 inhabitants than larger cities. Searches were seasonally stable with a Germany-wide peak in July 2017. This study provides an overview of public interest in axSpA based on web search data in Germany. The identified search patterns could be used to guide public health campaigns and optimize axSpA management in Germany.

**Supplementary Information:**

The online version contains supplementary material available at 10.1007/s00296-023-05273-x.

## Introduction

Axial spondyloarthritis (axSpA) is a systemic rheumatic disease that predominantly affects the spinal and sacroiliac joints (SIJ) [1]. Symptoms usually occur in young adulthood and progress in flare-ups of pain, fatigue, and functional impairment [2]. Within a wide range of manifestations, the core symptom of axSpA is inflammatory back pain (IBP) [1]. However, while back pain is a widespread and usually nonspecific health complaint [3], axSpA is a biopsychosocial condition, leading to disability and psychological, social, and occupational distress [4, 5].

The broad clinical spectrum of axSpA is historically divided into a radiographic (r-axSpA) and a non-radiographic (nr-axSpA) subset [6]. R-axSpA, also known as ankylosing spondylitis (AS) or “Morbus Bechterew” in Germany [7], is defined by the presence of radiographic sacroiliitis, whereas nr-axSpA is a newer concept, acknowledging that patients can also be diagnosed with normal X-ray imaging based on clinical patterns and magnetic resonance imaging [6].

The prevalence of axSpA is associated with the genetic distribution of the human leukocyte antigen (HLA) B27 [1]. However, as classification criteria have changed considerably over time, epidemiologic data are inconsistent and mostly restricted to r-axSpA [8]. In Germany, an AS prevalence of 0.3–0.5% is assumed [7], while for nr-axSpA, no reliable prevalence data are available [8]. A high number of undiagnosed and inadequately treated individuals must be assumed [1] given the shortage of rheumatologists in Germany [9] and the mean diagnostic delay of approximately 7 years [4, 10]. Data collected in the traditional medical settings may therefore not capture the true burden of axSpA, for which population-based approaches may offer an unconventional alternative [11, 12].

In 2020, more than 90% of Germans used the Internet daily and more than 70% utilized it for health information [13]. Similarly, the internet was the most frequently used source of health information among German rheumatic patients in 2019, with 87% reporting that they had previously obtained health information online [14]. Web search analysis therefore enables a comprehensive spatiotemporal overview of the interests of the Internet-using population and is gaining momentum in medical research as well [11]: Previous work has shown that public online search behavior can forecast infectious disease outbreaks [15], correlate with media and environmental influences [11, 16, 17], and reveal topics of interest that patients are not comfortable sharing with their doctors [17]. Conventional clinical data remain essential. However, infodemiology can complement them by providing population-based data that also include people and situations outside of the medical setting [11]. This is particularly important to better understand underdiagnosed conditions like axSpA. This study employs an infodemiologic approach to elucidating public interest, unmet needs, and overall burden of axSpA in Germany by analyzing thematic, geographic, and temporaI patterns in web search data.

## Methods

### Study design and data extraction

A 4 year retrospective analysis of the online search volume (SV) related to axSpA in Germany between January 2017 and December 2020 was performed using Google Ads Keyword Planner. Although this tool is primarily intended for marketing purposes, it has proven to be a valuable source of data in the scientific field of infodemiology by identifying relevant keywords/phrases for a given topic along with their estimated search frequency [11]. To investigate interest in axSpA, an initial sample of six representative keywords was selected and entered into the tool: “axial spondyloarthritis”, “axSpA”, and “Morbus Bechterew” (common German synonym for AS) were selected as stand-alone terms for the disease. The other keywords were selected to also consider affected individuals who might not have an official axSpA diagnosis or are unfamiliar with the terminology but are nevertheless interested in investigating the disease and its core symptom: “rheumatism and back pain”, “rheumatic back pain”, and “inflammatory back pain”. The AI-based tool then identified additional relevant keywords and their monthly search frequency for the past 48 months. The settings were set to only include search queries from Google users whose IP-address and language preference were German. Data were obtained for the whole of Germany and 16 major cities distributed across the country (Berlin, Cologne, Dortmund, Dresden, Düsseldorf, Erfurt, Freiburg, Hamburg, Hannover, Kiel, Magdeburg, Munich, Nuremberg, Rostock, Saarbrücken, and Stuttgart). Web search data for Germany as a whole were used for a detailed content analysis. A comparison of cities was added to explore regional differences in search behavior, thus creating a more nuanced picture of axSpA search interest in Germany.

### Content categorization

**Fig. 1:**
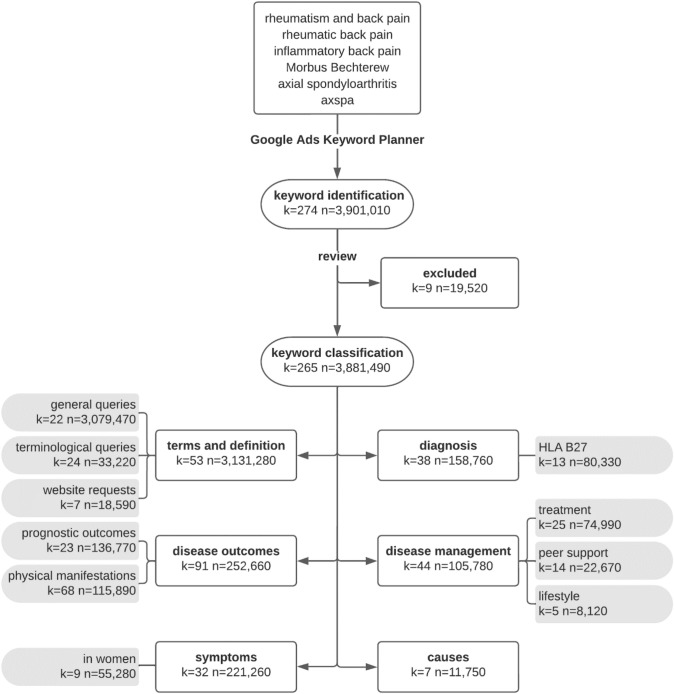
Flowchart of data extraction and content categorization process

All identified keywords were reviewed individually, and nine keywords that were irrelevant to axSpA or its pathology were excluded from the analysis (e.g., “back pain due to bacteria”, “morbus behçet life expectancy”). After qualitatively assessing the remaining keywords, six content categories were defined inductively: (1) *terms and definition* (e.g., “spondyloarthritides”, “rheumatism Morbus Bechterew”), (2) *disease outcomes* (e.g., “life expectancy”),  (3) *disease management* (e.g., “treatment”, “experience reports”),  (4) *diagnosis* (e.g., “Bechterew diagnosis”, “HLA B27 positive”), (5) *symptoms* (e.g., “rheumatism back symptoms”), and (6) *causes* (e.g., “Morbus Bechterew genes”, “psychological causes”). Each keyword was qualitatively assigned to one category. Recurring themes within a category were further clustered into subcategories (e.g., *HLA B27*, Fig. 1).

### Statistical analysis

Descriptive data were generated for all categories. To compare the SV of the selected cities, the SV was calculated per 100,000 inhabitants (2019) [18] and one-way analysis of variance (ANOVA) with a Dunn–Bonferroni post hoc test was performed. Whenever the assumptions for ANOVA were not met, Kruskal–Wallis test with a Dunn–Bonferroni post hoc test was used. Seasonal patterns were evaluated through (partial) autocorrelation function (PACF). IBM SPSS Statistics for Windows, Version 24.0 (IBM Corp, Armonk, NY) was used for the statistical analysis.

## Results

### Content analysis

A total of 265 keywords related to axSpA with a SV of 3,881,490 searches were identified (Fig. 1). “Morbus Bechterew” was the most commonly searched keyword (*n* = 2,357,000). Overall, 206 additional keywords contained “(Morbus) Bechterew” (e.g., “Morbus Bechterew symptoms”), whereas technical terms like “ankylosing spondylitis” and “axial spondyloarthritis” were only found in 24 keywords (e.g., “spondyloarthritis symptoms”).

Around 80.7% of the total SV were assigned to the category *terms and definition* and only 0.3% referred to *causes*. While the category *terms and definition* (*n* = 3,131,280) mainly consisted of *general queries* (98.3%; *n* = 3,079,470; e.g., “Morbus Bechterew”, “axial spondyloarthritis”), other categories revealed particular areas of interest (Fig. 1). For example, 50.6% of searches for *diagnosis* (*n* = 158,760) referred to *HLA B27*, and 25.0% of searches for *symptoms* (*n* = 221,260) referred specifically to *women*. Among *disease management* (*n* = 105,780), 70.9% of the searches focused on *treatment*, whereas 21.4% focused on *peer support*. The category *disease outcomes* (n = 252,660) was subdivided into *physical manifestations* (45.9%) and *prognostic outcomes* (54.1%).

**Fig. 2:**
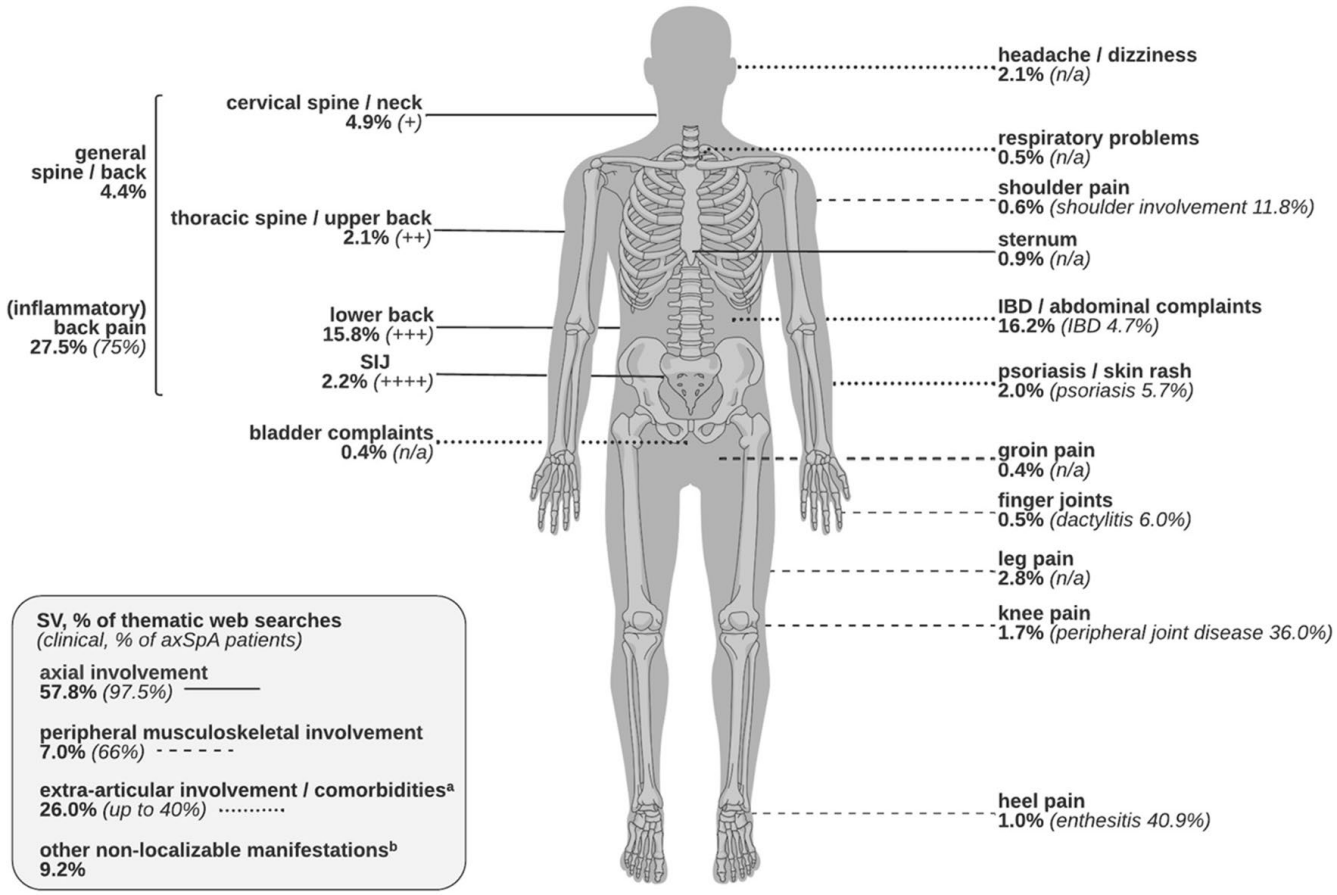
Population-based searches of axSpA disease outcomes in Germany identified through web search data from January 2017 to December 2020: *Physical manifestations* indicated with their search volume (% of all searches for *physical manifestations*) and corresponding clinical prevalence (% of affected axSpA patients) [7, 19]: **a** also including non-localizable and general searches for comorbidities (4.8%, e.g., “comorbidities in Morbus Bechterew”) in addition to the localizations visualized in the scheme (21.2%). **b** e.g., “Morbus Bechterew pain”

**Fig. 3:**
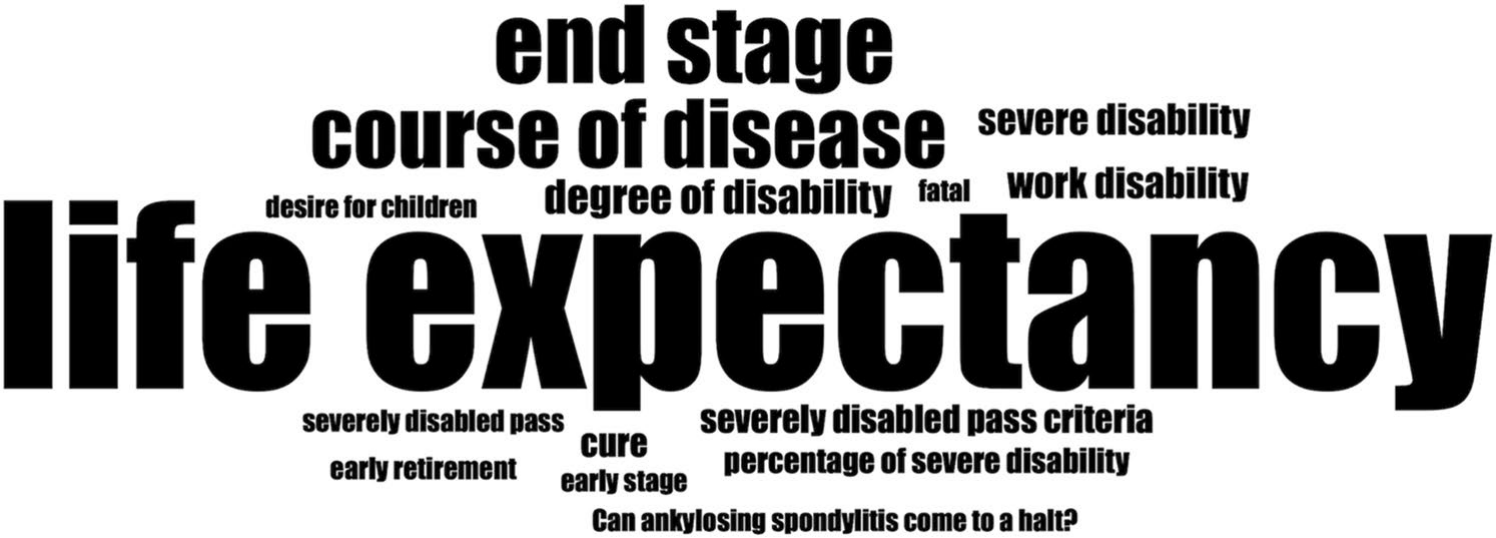
Population-based searches of axSpA disease outcomes in Germany identified through web search data from January 2017 to December 2020: *Prognostic outcomes* (font size adapted to search frequency)

Among *physical manifestations*, nearly half of the keywords contained “pain” (*n* = 52,880; 45.6%), and the most searched manifestation sites were axial localizations (e.g., spine, back, neck, and SIJ; *n* = 66,930, 57.8%) followed by abdominal comorbidities (e.g., inflammatory bowel disease (IBD); *n* = 18,750, 16.2%) (Fig. 2). However, there were 18.0% more searches focusing on *prognostic outcomes* than on *physical manifestations*. The most searched prognostic concern was “life expectancy” (*n* = 67,440; 49.3%). Other searches for *prognostic outcomes* are displayed in Fig. 3, ranging from pragmatic (e.g., “severely disabled pass criteria”, *n* = 2,650, 1.9%) to drastic (e.g., “Morbus Bechterew fatal”, *n* = 760, 0.6%).

### Comparison of cities

During the study period, 4667 searches per 100,000 inhabitants were measured in the whole of Germany and thus substantially less than in the examined cities (average of 8935 searches/100,000 inhabitants, Table 1). The analysis showed that larger cities tended to have a lower SV per 100,000 inhabitants than less populated cities. For example, Berlin had the lowest monthly SV per 100,000 inhabitants (median: 145.0, interquartile range: [135.8; 154.2]), whereas Freiburg had the highest (285.5 [262.8; 307.1]; *p* < 0.001). Furthermore, the proportion of the overall SV referred to *terms and definition* was generally higher in larger cities like Berlin (70.5%) than in smaller cities like Saarbrücken (52.3%, Table 1). For each category, significant differences in SV were identified between the cities.

**Table 1:** Web search volume related to axSpA in Germany from January 2017 to December 2020

City^a^	Number of inhabitants in 2019	Number of searches per 100,000 inhabitants	Proportional distribution of searches among content categories, %					
			Terms and definition^b^	Outcomes^c^	Symptoms^c^	Diagnosis^c^	Management^c^	Causes^c^
Berlin	3,669,491	7054	70.5	11.3	6.3	5.9	5.1	0.9
Hamburg	1,847,253	8452	67.0	12.9	6.4	6.4	6.3	1.0
Munich	1,484,226	9055	67.7	12.4	6.1	6.6	6.2	1.0
Cologne	1,087,863	9464	63.8	14.0	6.9	7.2	7.0	1.1
Stuttgart	635,911	10,237	60.8	15.5	7.4	7.8	7.4	1.1
Düsseldorf	621,877	8812	57.8	15.8	8.2	8.7	8.6	0.9
Dortmund	588,250	9527	56.7	16.3	9.0	8.3	8.2	1.5
Dresden	556,780	10,234	59.9	14.7	8.6	7.6	8.1	1.2
Hannover	536,925	10,845	59.6	15.6	8.1	7.4	8.1	1.3
Nuremberg	518,370	9912	58.9	14.9	8.5	8.5	8.1	1.0
Kiel	246,794	11,374	55.7	16.1	10.2	9.7	7.6	0.7
Magdeburg	237,565	10,250	54.4	15.3	10.8	9.4	8.8	1.3
Freiburg	231,195	13,781	59.2	14.0	8.6	8.6	8.7	0.9
Erfurt	213,981	10,926	53.3	17.6	10.6	8.9	8.3	1.4
Rostock	209,191	12,209	55.5	15.5	9.9	9.8	7.6	1.7
Saarbrücken	180,374	11,909	52.3	17.4	10.9	10.1	8.3	1.1
Urban average	804,128	8935	63.6	13.7	7.4	7.3	6.9	1.0
Germany	83,166,711	4667	80.7	6.5	5.7	4.1	2.7	0.3

Comparison of the numbers of searches per 100,000 inhabitants across Germany and in 16 major cities considering population size and content categorization

^a^Cities sorted by descending population size

^b^ANOVA and Bonferroni post hoc test

^c^Kruskal–Wallis and Dunn–Bonferroni post hoc test

### Time course

**Fig. 4:**
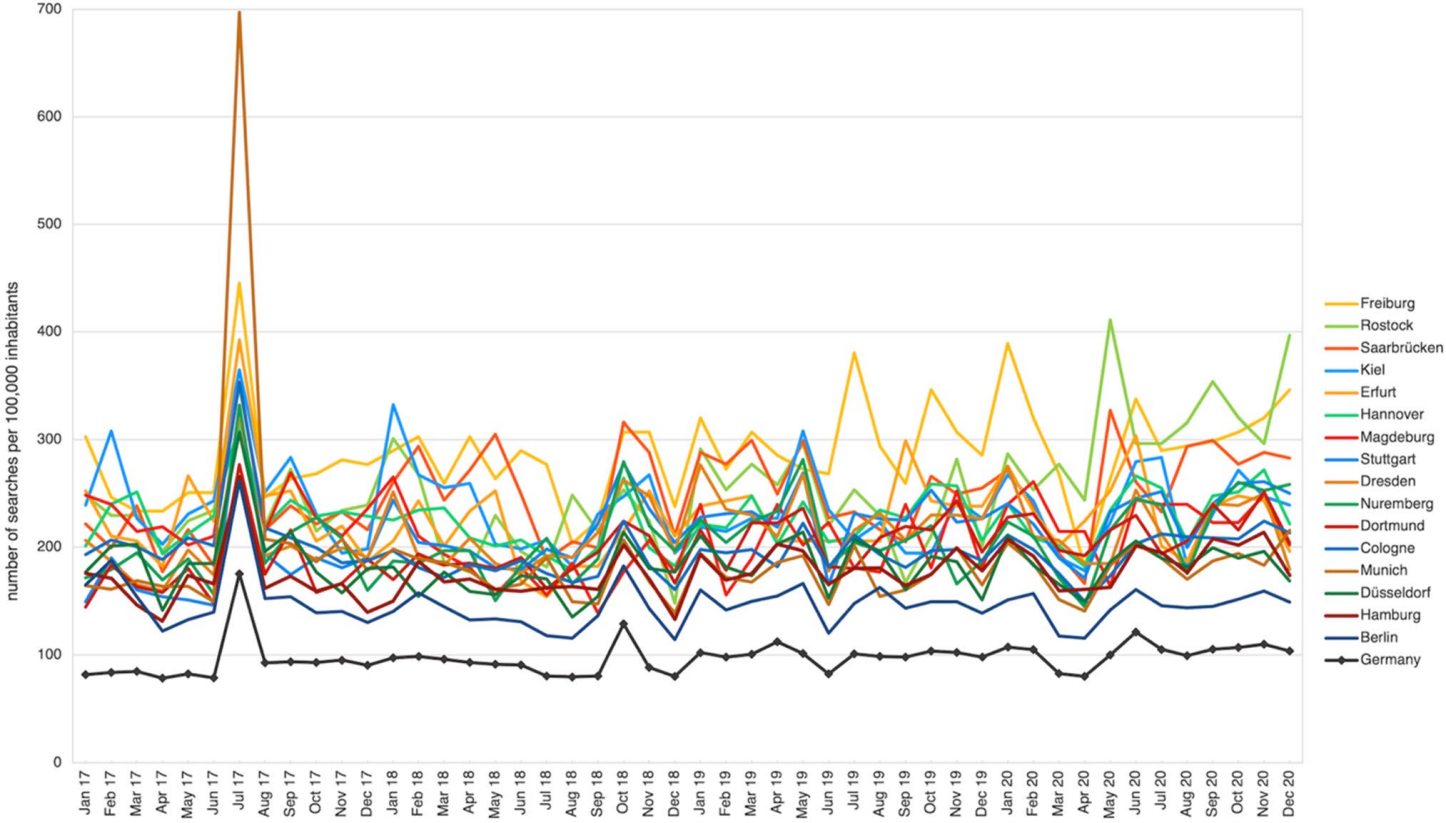
Time course of axSpA-related web searches from January 2017 to December 2020 in 16 German cities and nationwide

In Germany, the number of searches was relatively stable over time with an average of 97.2 ± 16.1 searches per 100,000 inhabitants per month (Fig. 4). A slight increasing trend in SV over the 4 years was observed, but no seasonal patterns were detected through PACF (Supplementary Fig. 1).

Smaller cities tended to show a higher overall SV and greater monthly fluctuations than larger cities (Fig. 4). There were a few peaks in SV Germany-wide, like those in July 2017 (*n* = 175 searches/100,000 inhabitants), October 2018 (*n* = 129 searches/100,000 inhabitants), and June 2020 (*n* = 121 searches/100,000 inhabitants). Except for Rostock and Saarbrücken, July 2017 was the month with the highest SV in each city. The peak in July 2017 was primarily due to an increase in *general queries* like the term “Morbus Bechterew” (increase by 172%). As demonstrated in Fig. 4, the peak was most prominent in Munich, where the SV increased by 366%.

## Discussion

In total, nearly 4 million search queries for axSpA were identified, of which 81% referred to *terms and definition*. The SV across the whole of Germany was comparatively lower than that of individual cities, and smaller cities generally had a higher SV than larger cities. Searches were seasonally stable, with a German-wide peak in July 2017.

High search interest was for general disease and treatment information, which is in line with previous research [20, 21]. Particularly, the genetic trait HLA B27 was searched very often. Since German guidelines recommend the determination of HLA B27 in patients with IBP [7], affected individuals may have been confronted with this laboratory value in their diagnostic process and initiated further research about it. Another notable proportion of the SV referred to women, whereas axSpA was long thought to be a predominantly male disease [22]. Recent research found that the ratio of men and women affected by axSpA evens out when non-radiographic forms are included [1]. Gender differences do exist, however, when considering symptoms and burden of axSpA; there is evidence that women tend to show less typical disease manifestation, are diagnosed on average 2 years later, and have poorer quality of life [22, 23]. The high number of searches addressing women may be the result of growing public and scientific interest, but it may also suggest an unmet need for information specifically regarding affected women.

IBP is the core symptom of axSpA [1, 7, 24]. Similarly, “pain”, especially “back pain”, was the most frequent keyword in the subcategory *physical manifestations*. Contrary to clinical data, intestinal comorbidities like IBD were most searched for among extra-articular manifestations and thus strongly overrepresented [19, 25]. Unspecific abdominal issues are both a common complaint in the general population [26] and an uncomfortable topic to discuss openly, which can result in increased online research [17]. However, considering that previous studies found microscopic gut inflammation in more than half of axSpA patients [27, 28], the results could also imply an actual underdiagnosis of early inflammatory bowel changes.

The emphasis of search interest, however, was on psychosocial aspects of axSpA rather than physical complaints: there was a notable number of searches about *peer support*, and there were twice as many searches about “life expectancy” than about “back pain”.

While some of the searches on *prognostic outcomes* addressed realistic outcomes like work disability, others implied severe concerns like reduced life expectancy or fatality, potentially reflecting fear of disease progression (FOP). FOP is defined as a realistic anxiety and frequent emotional strain in chronically ill patients that impairs quality of life [29]. Most studies investigating mental health in axSpA focused on depression and anxiety disorders [5, 30]. However, this and other studies indicated that FOP may also be a notable mental health risk in axSpA. For example, Berg et al. observed that out of 11 chronic diseases, rheumatic diseases scored highest for FOP [29]. Furthermore, Garrido-Cumbrera et al. described that the most-stated fear among individuals with axSpA was FOP [4].

Comparable to previous web search analyses [20, 31], this study found that the SV per 100,000 inhabitants was nearly twice as high in cities as in the whole of Germany, with smaller cities having a considerably higher SV. Previous analyses found positive correlations between the Google SV and the number of specialists [17] or the respective incidence rates [20, 32]. It was not possible to calculate correlations in this study, because prevalence data are inconsistent [8], and no comparable data on rheumatologic medical supply in German cities are available [33].

Some studies also established a link between web search frequency and environmental parameters [11, 17, 32]. Changes in the annual search behavior were observed for different chronic non-communicable diseases like psoriasis [16] or sarcoidosis [31]. Although weather sensitivity is also a frequent complaint in rheumatology [34], there is no clear evidence how strongly rheumatic pain is linked to weather conditions [34–36]. This study, like a recent analysis on AS [37], found no seasonality in web search interest in axSpA. Thus, the study results do not suggest a general effect of seasonal weather conditions on the burden of axSpA as observed in other chronic conditions. There was, however, a sharp peak in SV in July 2017, which may have been influenced by media reports [11, 38]. In July 2017, the German tabloid press reported extensively on the diagnosis of a famous actress with ankylosing spondylitis [39]. This association is illustrated by the fact that the newspaper mainly used the term “Morbus Bechterew”, which was the term with the largest increase that month.

### Limitations

In contrast to personal consultations, online search behavior is less influenced by feelings of shame or social desirability [17]. However, other sources of bias might arise, such as the automatic completion of keywords suggested by the search engine or the approximations of monthly searches provided by the Keyword Planner. With Google being the primary search engine for most Internet users in Germany [17], this study aims to capture the interests of a comprehensive part of the German population. However, the observed search behavior could be distorted by only including people using the search engine in German language. Larger cities tend to have larger international non-German speaking communities than smaller cities, which could be an explanation for the observed differences. Furthermore, without any user demographics provided, it is unclear to what extent the searches originated from affected individuals and their relatives or from other interest groups in the population, such as healthcare professionals or medical students looking up information on axSpA online for vocational purposes.

### Conclusions

Web search data enable insight into public interests, which can benefit both individual patient care and public health measures. This analysis found that prognostic concerns, like life expectancy, generated a higher SV than physical manifestations, like pain. Thus, the study underlines the psychosocial burden of axSpA, implying an unmet need for a holistic approach in patient care. Unmet needs may vary by cultural context and country and could easily be elucidated by web search analyses in different languages and countries. Regional and temporal analysis suggested an important role of media on public search interest. This influence could also be leveraged to provide evidence-informed health campaigns. By complementing clinical data with web search data, valuable synergies could be created with the goal of earlier diagnosis, better information availability, and an overall decrease of the burden of axSpA.

## Supplementary Information

Below is the link to the electronic supplementary material.

Supplementary file1 (DOCX 143 kb)

## Data Availability

The raw data supporting the conclusions of this article will be made available by the authors upon request.
