# Supplementary material for: Analyzing web searches for axial spondyloarthritis in Germany: a novel approach to exploring interests and unmet needs
Source: Rheumatol Int. 2023 Jan 14;43(6):1111–9. doi: 10.1007/s00296-023-05273-x (PMC10125933; doi:10.1007/s00296-023-05273-x)
Supplement: Supplementary file 1 [file 296_2023_5273_MOESM1_ESM.docx]

Supplementary Figure 1. Partial autocorrelation function plot (PACF)[a] for the monthly number of web searches related to axSpA (with 5% significance limits for the partial autocorrelations).

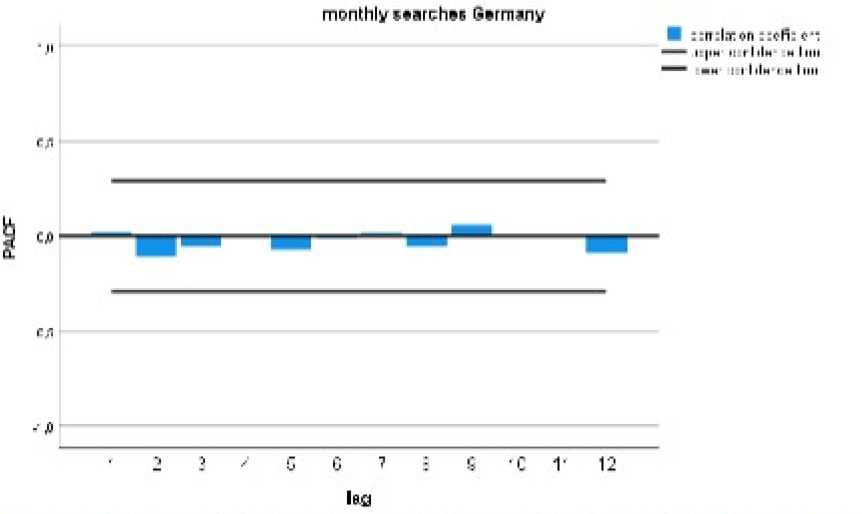

[a] Measure of the correlation between values of a time series that are k intervals apart, adjusted for the terms of the intervening intervals. X-axis: indicates lag k at which the autocorrelation is calculated. Y-axis: indicates the value of the correlation [-1;1]. A peak at lag k extending beyond the significance limits indicates a significant correlation between the number of searches in month m and the number of searches that occurred m-k months earlier. This PACF plot, with a total of 12 lags (annual seasonality), shows no significant autoregressive terms within the monthly searches.
